# *Amblyomma hebraeum* is the predominant tick species on goats in the Mnisi Community Area of Mpumalanga Province South Africa and is co-infected with *Ehrlichia ruminantium* and *Rickettsia africae*

**DOI:** 10.1186/s13071-020-04059-5

**Published:** 2020-04-21

**Authors:** Frans Jongejan, Laura Berger, Suzanne Busser, Iris Deetman, Manon Jochems, Tiffany Leenders, Barry de Sitter, Francine van der Steen, Jeanette Wentzel, Hein Stoltsz

**Affiliations:** 1 Vectors and Vector-borne Diseases Research Programme, Department of Veterinary Tropical Diseases, Faculty of Veterinary Science, University of Pretoria, Private Bag X04, Onderstepoort, 0110 Republic of South Africa; 2 Utrecht Centre for Tick-borne Diseases (UCTD), FAO Reference Centre for Ticks and Tick-borne Diseases, Faculty of Veterinary Medicine, Utrecht University, Yalelaan 1, 3584 CL Utrecht, The Netherlands; 3 Present Address: Small Animal Hospital, Royal (Dick) School of Veterinary Studies, University of Edinburgh, Easter Bush Campus, Edinburgh, EH25 9RG UK; 4 Present Address: Cape Cross Veterinary Hospital, 8 Jacaranda Street, Wavecrest, Jeffrey’s Bay, 6330 Republic of South Africa; 5 Hans Hoheisen Research Platform, Center for Veterinary Wildlife Studies, Faculty of Veterinary Science, University of Pretoria, Hans Hoheisen Wildlife Research Station, Orpen, Mpumalanga Republic of South Africa

**Keywords:** *Amblyomma hebraeum* ticks, South Africa, *Ehrlichia ruminantiu*m, Heartwater, *Rickettsia africae*, Tick bite fever

## Abstract

**Background:**

In sub-Saharan Africa, *Amblyomma* ticks are vectors of heartwater disease in domestic ruminants, caused by the rickettsial pathogen *Ehrlichia ruminantium*. Immature tick stages often bite humans, whereby they act as vectors of tick-bite fever caused by *Rickettsia africae.* Moreover, *Amblyomma* ticks cause damage to livestock due to their feeding behaviour. In South Africa, we studied the abundance of *Amblyomma hebraeum* ticks on goats of emerging farmers in Mpumalanga Province. A selected number of *A. hebraeum* nymphs and adult ticks was tested for co-infection with *E. ruminantium* and *R. africae*.

**Methods:**

A total of 630 indigenous goats, belonging to farmers in the Mnisi Community area, were examined for ticks in 2013 and 2014. All ticks were identified, and a selected number was tested by PCR with reverse line blot hybridisation.

**Results:**

In total, 13,132 ticks were collected from goats distributed over 17 different households. *Amblyomma hebraeum* was the predominant species, followed by *R. microplus*. *Rhipicephalus appendiculatus*, *R. simus* and *R. zambeziensis* were also identified. *Amblyomma hebraeum* was present throughout the year, with peak activity of adults in summer (November) and nymphs in winter (July). The ratio between adults and nymphs ranged from 1:2.7 in summer to 1:55.1 in winter. The mean prevalence of infection for *E. ruminantium* by PCR/RLB in adult ticks was 17.4% (31/178), whereas 15.7% (28/178) were infected with *R. africae*. In pooled nymphs, 28.4% were infected with *E. ruminantium* and 38.8% carried *R. africae* infection. Co-infections of *E. ruminantium* and *R. africae* in adult and pooled nymphal ticks were 3.9% (7/178) and 10% (14.9), respectively. Lameness of goats due to predilection of ticks for the interdigital space of their feet was observed in 89% of the households.

**Conclusions:**

Goats act as important alternative hosts for cattle ticks, which underscored the necessity to include goats in control programs. It is suggested to use acaricide-impregnated leg-bands as a sustainable method to kill ticks and prevent lameness in goats. The challenge of goats by considerable numbers of *E. ruminantium-*infected ticks is a major obstacle for upgrading the indigenous goat breeds. Humans may be at risk to contract tick-bite fever in this area.
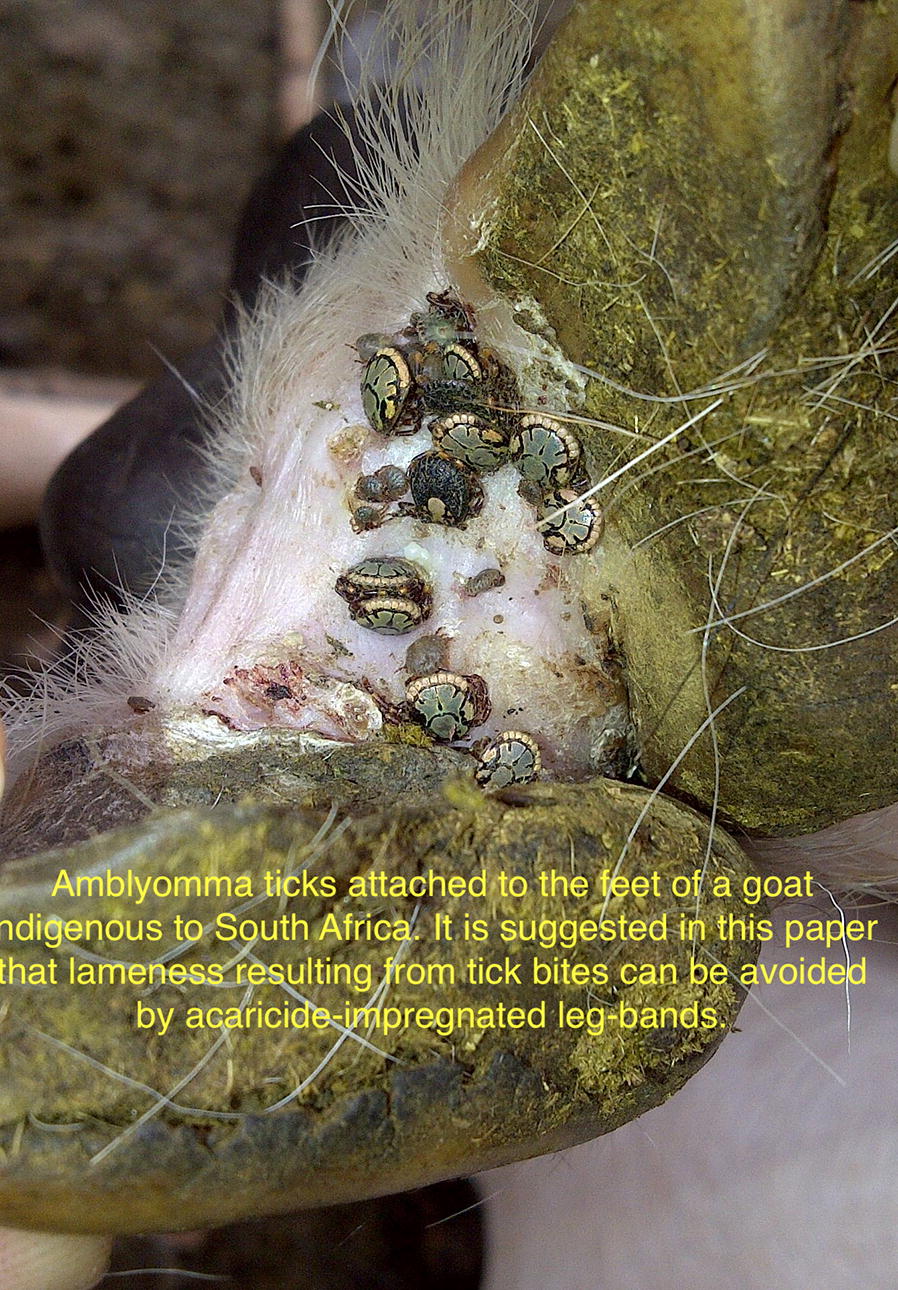

## Background

Ticks and tick-borne diseases constitute a major cause of economic loss to the livestock sector, in particular with respect to the production of cattle and small ruminants in tropical and sub-tropical areas [1]. Ticks are also of great importance to companion animals, livestock and humans due to their capacity to transmit a broad range of bacterial, protozoan and viral pathogens [2, 3]. In sub-Saharan Africa, ixodid ticks, in particular *Amblyomma* species, are of significant concern regarding their ability to transmit *Ehrlichia ruminantium.* This rickettsial pathogen belongs to the family *Anaplasmataceae* and is widely distributed throughout sub-Saharan Africa, where it causes heartwater disease in cattle and in small ruminants [4]. Another rickettsial pathogen also transmitted by *Amblyomma* ticks is *Rickettsia africae*, which is the cause of African tick-bite fever in humans [5]. Tick-bite fever is a well-known disease affecting travellers to sub-Saharan Africa and characterised by fever, multiple eschars and maculopapular skin rashes, but is usually resolved after doxycycline therapy [6–8].

In southern Africa, indigenous goats play an important economic and cultural role in the livelihoods of the rural farming communities [9]. However, cattle create more value and status in these communities and are therefore usually better cared for than goats. Nevertheless, one needs to keep in mind that goat production efficiency cannot be measured in saleable livestock numbers only, since raising goats is considered an insurance against emergencies by these farmers. Farmers’ perception of mortality among young goats under communal farming conditions in South Africa has been surveyed, whereby ectoparasites (predominantly ticks) scored high and were perceived a major cause of mortality [10].

*Amblyomma hebraeum*, also known as the South African bont tick, is a notorious tick infesting cattle, sheep and goats as well as a range of wildlife species; it is also the local vector of heartwater disease. *Amblyomma hebraeum* is exclusively a southern African tick and occurs in the coastal regions of the Eastern Cape and KwaZulu-Natal provinces in South Africa as well as in southern Mozambique. Its distribution extends inland through Swaziland, the Mpumalanga, Limpopo and North West provinces of South Africa into eastern Botswana to south-western Zimbabwe [11, 12]. Tick infestation on cattle can lead to the loss of udder quarters, whereas scrotal tick damage has recently been linked to infertility in communal bulls in the North West Province in South Africa [13]. In goats, skin lesions, in particular in the inter-digital space (a predilection site for the ticks) are prone to secondary infections and often result in lameness. The association between foot abscesses in goats and the relationship with adult *A. hebraeum* ticks has already been known for more than 30 years [14].

At a farmers’ day organised in the Mnisi Community area, farmers confirmed that they are often confronted with lameness of their goats due to tick infestation. Moreover, owners indicated that they have to cope with losses when goats die suddenly or after displaying leg-pedalling movements prior to death. The latter is an indication that some of their goats acquire a fatal *E. ruminantium* infection transmitted by *Amblyomma* ticks and succumb to heartwater disease, although a definitive diagnosis is usually not made.

Besides ticks of the genus *Amblyomma*, insight into the overall species composition and distribution of ticks infesting goats is relevant. Ticks found on goats may play a role in the epidemiology of cattle diseases and may carry zoonotic pathogens [15]. It has been reported that tick species other than *A. hebraeum*, commonly infesting goats in the Eastern Cape Province and in Maputo Province, were *Rhipicephalus microplus*, *R. appendiculatus* and *R. evertsi* [11, 15]. One tick of particular importance is the Asian blue tick, *R. microplus*. This tick primarily uses cattle as hosts and is usually only found on other animals provided infested cattle are present at the same location. Finding *R. microplus* on goats therefore indicates that this species is in the process of adapting to goats [15]. On cattle, in the Eastern Cape Province and Maputo Province, displacement of the indigenous tick *R. decoloratus* by the introduced species *R. microplus* has already taken place [16].

In the Mnisi Community area, situated in Mpumalanga Province in close proximity to the southern part of the Kruger National Park, surveys of ticks on small ruminants have not been carried out. Also, displacement of *R. decoloratus* by *R. microplus* may have occurred in this area, but this needs to be confirmed. As the study area borders on wildlife areas, and *R. microplus* is known to feed on wildlife hosts, the presence or absence of this species on goats may be of significance.

In this context, the aim of this study was to investigate the relative abundance of ticks and their impact on indigenous goats owned by emerging farmers in the Mnisi Community area as a basis for a sound intervention strategy to reduce the direct and indirect damage caused by ticks. Information on the species composition of ticks infesting goats will provide a better understanding of the potential transmission of tick-borne pathogens affecting local livestock and the possible risks for humans to acquire zoonotic tick-borne diseases.

## Methods

### Study area

**Fig. 1:**
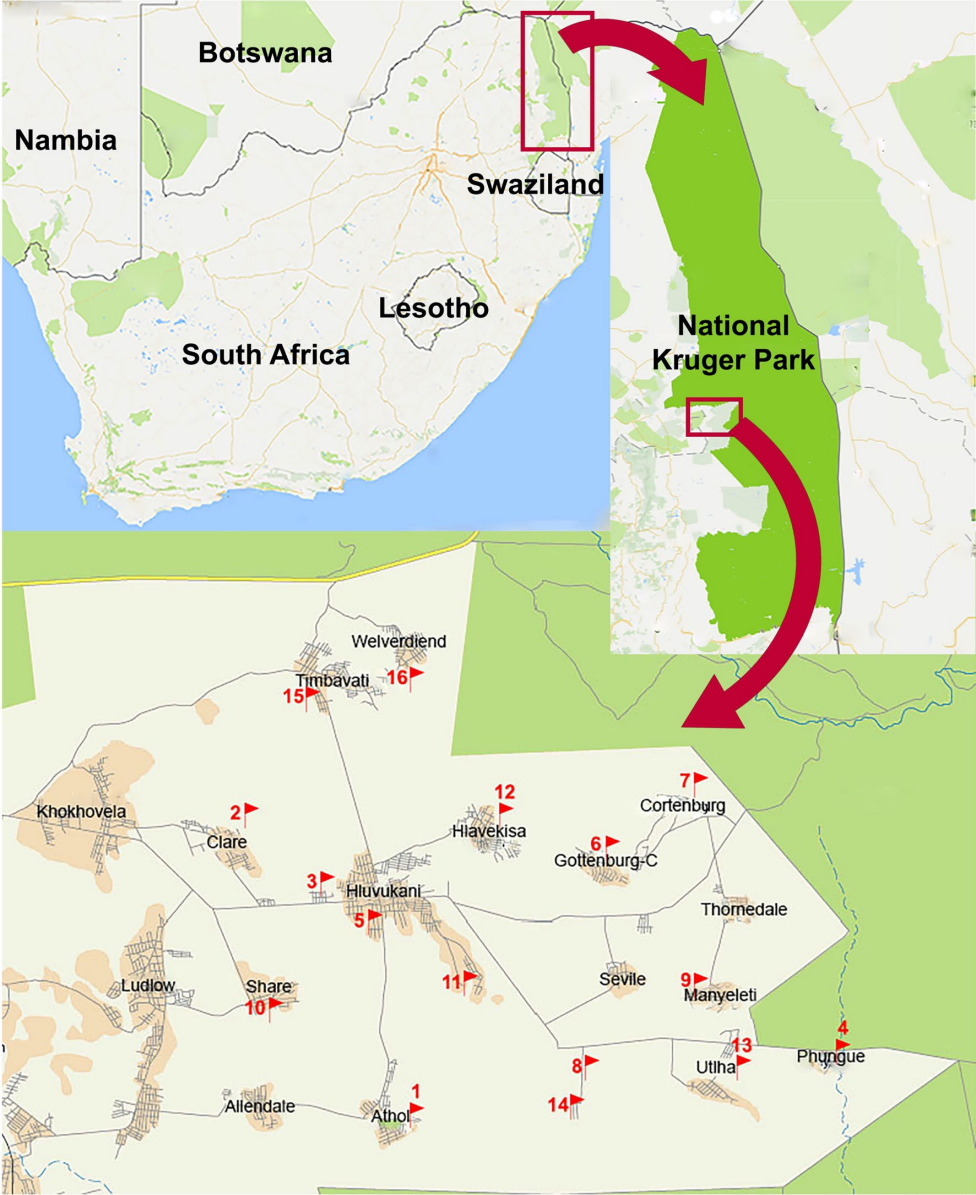
Geographical location of the Mnisi Community study area in Mpumalanga Province of South Africa

The study area encompassed households in 17 villages in the area of Mnisi, province of Mpumalanga, South Africa (Fig. 1). This area covers about 29,500 hectares and is situated in the northeastern corner of the Bushbuckridge Municipal Area within a typical savannah ecosystem. It is surrounded by the adjacent Andover and Manyeleti provincial game reserves as well as by the Kruger National Park (Fig. 1). The Mnisi community consists of over 40,000 people, divided over an estimated 8555 households, of which 917 different goat farmers own a total of approximately 6000 goats. The area is part of the Mnisi Community Programme, an initiative by the University of Pretoria and the Mnisi Traditional Authority. Dip-tanks (numbers 1–16) are operational for cattle in or near all 17 villages in the Mnisi area at no cost to the farmers (Fig. 1). Seventeen households located in or near the villages were included in the study.

### Study design

In both collections conducted in 2013, the number of goats varied considerably, whereas the collections in 2014 could be standardised to 10 animals per household. Goats were randomly selected in the morning in their kraals prior to going out for grazing. In total, 630 goats were examined for ticks by conducting whole body collections. Ticks were stored in collection tubes containing 70% ethanol. Each tube was labelled with the date, household and predilection site on the goat. The adult ticks were identified to the species level, whereas the nymphs were separated according to the genus under a stereoscopic microscope using standard taxonomic keys. Larvae from each collection tube were examined under the microscope to confirm the presence of *Amblyomma*. The mean number of *A. hebraeum* ticks per goat collected in each sampling period was statistically analysed by using a two-tailed, Mann-Whitney U-test.

### DNA extraction

*Amblyomma hebraeum* ticks were placed in sterile 2 ml microcentrifuge tubes containing 180 μl of a lysis buffer (GeneJet genomic DNA purification kit, Thermo Fisher Scientific, Landsmeer, the Netherlands) and were frozen at − 20 °C. Adult ticks were tested individually, nymphs in pools of ten ticks. Thereafter, metal beads (5 mm in diameter) were added to the frozen samples, which were disrupted in a TissueLyser (Qiagen Benelux BV, Venlo, the Netherlands) for 3 min at 50 Hz. The DNA was extracted from the triturated samples by using a GeneJet genomic DNA purification kit (Thermo Fisher Scientific, Landsmeer, the Netherlands) according to the instructions of the manufacturer. Extracted DNA was eluted in 150 μl elution buffer and used directly or stored at − 20 °C. After extraction, DNA was PCR amplified and tested by reverse line blot hybridisation (RLB).

### PCR

For *Anaplasma/Ehrlichia* and *Rickettsia* PCR, the primer pair Ehr-F2 (5’-AGA GTT TGA TCC TGG CTC AG-3’) and Ehr-R2 (5’-biotin-GAG TTT GCC GGG ACT TYT TCT-3’) was used to amplify the V1 variable region from the *16S* rRNA gene [17]. The length of the PCR amplicon was 460–500 bp. Each PCR was performed in a volume of 20 μl, containing 10 μl of 2× Phusion Hot Start High Fidelity Master Mix (Thermo Fisher Scientific), 0.5 μM of each primer and 2 μl of extracted genomic DNA; the remaining volume was double-distilled water. The PCR primers were purchased from Life Technologies Europe BV, Bleiswijk, the Netherlands. As positive controls, genomic DNA from *Ehrlichia canis* was used. Distilled water was used as negative control.

### Reverse line blot (RLB) hybridisation

Reverse line blot (RLB) hybridisation has the advantage of enabling the analysis of multiple samples against multiple probes simultaneously and is used to differentiate *Anaplasma* and *Ehrlichia* species [17]. Probes for the differentiation of *Rickettsia* species were also added to the membrane. Oligonucleotide probes containing an N-terminal N-(trifluoracetamidohexyl-cyanoethyl-N,N-diisopropyl phosphoramidite [TFA])-C6 amino linker were synthesised by Thermo Fisher Scientific. Specific probes targeted eight *Ehrlichia/Anaplasma* species; in addition to one catch-all probe for *Ehrlichia/Anaplasma*, specific probes to differentiate *E. ruminantium* from a range of related species were included. These were *A. centrale*, *A. marginale*, *A. phagocytophilum*, *A. bovis*, *A. platys*, *E. canis* and *E. chaffeensis*. Finally, *R. conorii*, *R. helvetica*, *R. africae* and *R. raoultii*, as well as a catch-all probe for *Rickettsia* detection, completed the membrane [18].

The RLB hybridisation was conducted as described previously [19]. Briefly, Biodyne C membranes were activated using 16% (wt/wv) 1-ethyl-3-(3-dimethyl-amino-propyl) carbodiimide (EDAC) (Carl Roth GmbH, Karlsruhe, Germany) for 10 min, after which the oligonucleotide probes were linked covalently to the membrane in 0.5 M NaHCO_3_, using a mini-blotter. Thereafter, the membrane was inactivated in 100 mM NaOH, washed in 2× SSPE/0.1% SDS at 60 °C and subsequently stored in 20 mM EDTA of pH 8.0. In each RBL assay, 10 μl of PCR product were added to 150 μl of 2× SSPE/0.1% SDS after denaturing for 10 min at 100 °C, followed by immediate cooling down on ice. Denatured PCR products were than hybridised to the Biodyne C membrane for 60 min at 42 °C. Subsequently, membranes were washed twice in 2× SSPE/0.5% SDS for 10 min at 50 °C, incubated at 42 °C for 30 min in 2× SSPE/0.5% SDS with 5 μl of streptavidin-POD conjugate (Roche Diagnostics, Mannheim, Germany), washed twice in 2× SSPE/0.5% SDS at 42 °C for 10 min and finally washed in 2× SSPE at room temperature for 5 min. Detection was carried out *via* chemiluminescence, using Amersham ECL detection reagents (Roche Diagnostics, Mannheim, Germany).

## Results

### Tick collections

**Fig. 2:**
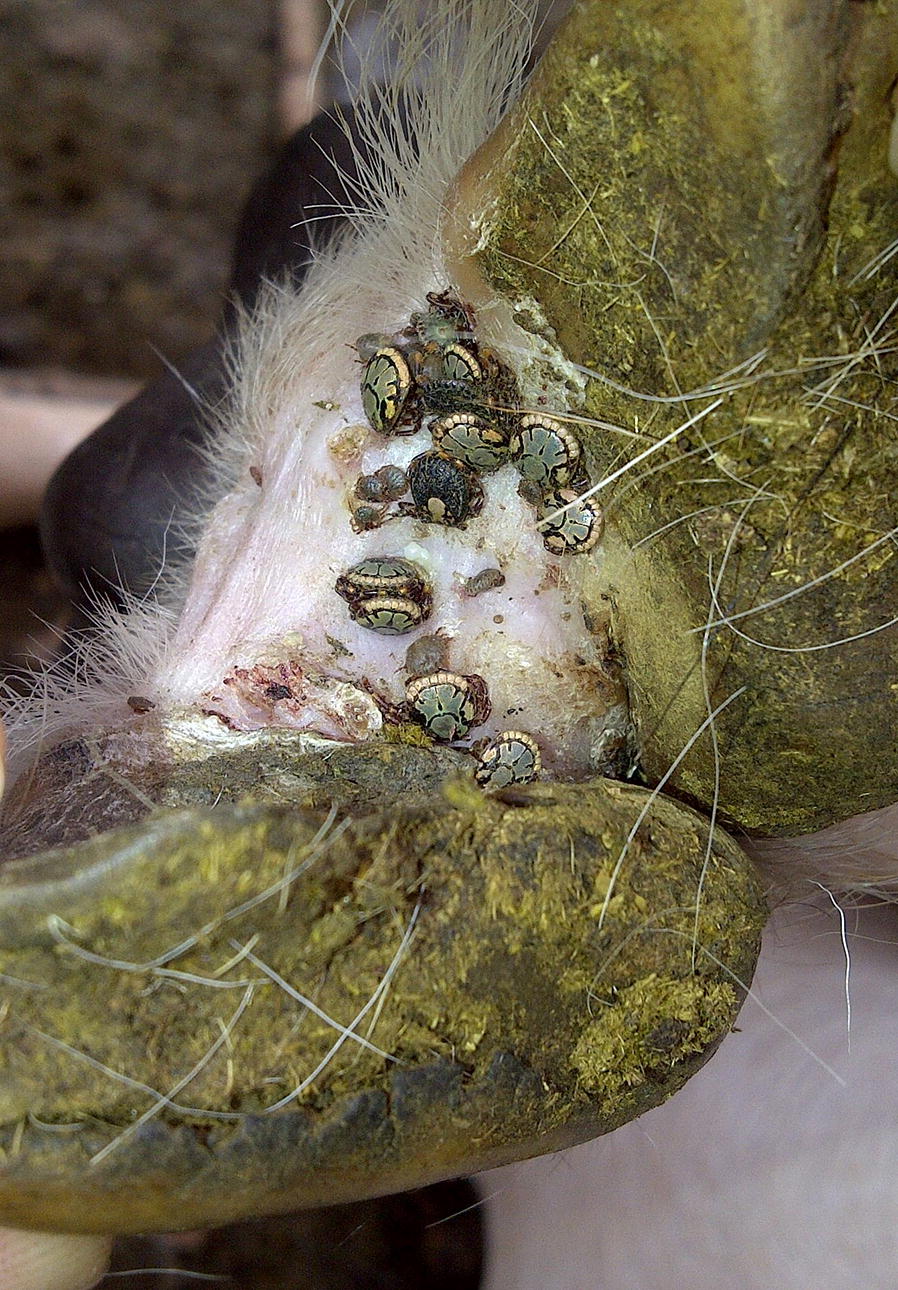
Infestation with adult *Amblyomma hebraeum* ticks in the interdigital space of the feet of an indigenous goat in the Mnisi Community Area

The tick species identified and the number of ticks collected from the goats are summarised in Tables 1, 2, 3 and 4. In total, 13,132 ticks were collected from goats distributed over 17 different households in the study area in Mpumalanga Province (Fig. 1). Five adult tick species were identified: *A. hebraeum*, *R. microplus*, *R. appendiculatus*, *R. zambeziensis* and *R. simus. Amblyomma hebraeum* was the predominant adult tick species, followed by *R. microplus* (Tables 1, 2, 3 and 4). The relative proportion of adult *Amblyomma* ticks *versus* the other tick species was 66.2%. A total of 4268 larvae was also collected from the goats. Adults as well as nymphal *A. hebraeum* ticks preferred to attach inside the interdigital space of the feet of goats, often leading to secondary infections and lameness, which was observed in 89% of the households (Fig. 2).

**Table 1:** Species composition and total number of ticks collected from goats in the Mnisi Community Area of Mpumalanga Province, South Africa, in July 2013

Village	No. of goats	Adults					Nymphs		Larvae	Total
		AH	RM	RA	RS	RZ	A	R		
Athol	11		3				21	2	236	262
Clare (H1)	3	1	16	1			21		60	99
Clare (H2)	3		1				32		77	110
Cortenburg	14						110	5	85	200
Gottenburg-C	2						14		2	16
Hlavakisa	8	9					132	3	62	206
Hluvukani (H1)	7	2	4				70		92	168
Hluvukani (H2)	14	5	1			1	50	22	13	92
Ludlow	8		11				38		55	104
Phungue	1	1	12				92	2	240	347
Share	11		2				35		77	114
Seville	3		1				48	11	0	60
Thorndale	4	1	1				36		38	76
Timbavati	14	1	71		1	1	90	23	108	295
Utha (H1)	4	3	30				327		650	1010
Utha (H2)	5		2				41	5	31	79
Welverdiend	5		6				37		106	149
Total	117	23	161	1	1	2	1194	73	1932	3387

*Abbreviations*: H1, Household 1; H2, Household 2; AH, *Amblyomma hebraeum*; RM, *Rhipicephalus microplus*; RA, *Rhipicephalus appendiculatus*; RS, *Rhipicephalus sinus*; RZ, *Rhipicephalus zambeziensis*; A, *Amblyomma*; R, *Rhipicephalus*

**Table 2:** Species composition and total number of ticks collected from goats in the Mnisi Community Area of Mpumalanga Province, South Africa, in November 2013

Village	No. of goats	Adults		Nymphs		Larvae	Total
		AH	RM	A	R		
Athol	10	13	3	102	2	48	168
Clare (H1)	7	32		16		17	65
Clare (H2)	10	34		51		5	90
Cortenburg	9	40		58	10	96	204
Gottenburg-C	19	67	7	88		41	203
Hlavekisa	15	71		304		85	460
Hluvukani (H1)	10	15		102		46	163
Hluvukani (H2)	14	17	4	20	3	9	53
Ludlow	6	6	11	114	2	24	157
Phungue	7	9		30		3	42
Share	5	48	4	17		3	72
Seville (H1)	13	16	4	62		66	148
Seville (H2)	10	45		97		68	210
Thorndale	13	37	1	156	1	97	292
Timbavati	9	22	1	67	1	10	101
Utha (H1)	7	33		50		46	129
Utha (H2)	7	9		68		28	105
Welverdiend	13	8		28		34	70
Total	184	522	35	1430	19	726	2732

*Abbreviations*: H1, Household 1; H2, Household 2; AH, *Amblyomma hebraeum*; RM, *Rhipicephalus microplus*; A, *Amblyomma*; R, *Rhipicephalus*

**Table 3:** Species composition and total number of ticks collected from goats in the Mnisi Community Area of Mpumalanga Province, South Africa, in March 2014

Village	No. of goats	Adults			Nymphs		Larvae	Total
		AH	RM	RA	A	R		
Athol	10	16	117	1	182	152	526	994
Clare (H1)	10	11	9	3	51	6	12	92
Clare (H2)	10	13	9		40	13	1	76
Cortenburg	10	5	1		55		3	64
Gottenburg-C	10	29	1		62	1	10	103
Hlavekisa	10	1			42	3	21	67
Hluvukani (H1)	10	1		3	254	2	58	318
Hluvukani (H2)	10	17	30		39	61	54	201
Ludlow	10	2	5	2	130	9	97	245
Phungue	10	19	4		49		1	73
Share	9	25	14	2	59	26	4	130
Seville	10	14	4	5	53	9	0	85
Thorndale	10	2			77		8	87
Timbavati	10	2	2	2	42	5	6	59
Utha (H1)	10	60	1	2	37			100
Utha (H2)	10	1	5	1	78	5	71	161
Welverdiend	10	1			22		6	29
Total	169	219	202	21	1272	292	878	2884

*Abbreviations*: H1, Household 1; H2, Household 2; AH, *Amblyomma hebraeum*; RM, *Rhipicephalus microplus*; RA, *Rhipicephalus appendiculatus*; A, *Amblyomma*; R, *Rhipicephalus*

**Table 4:** Species composition and total number of ticks collected from goats in the Mnisi Community Area of Mpumalanga Province, South Africa, in July 2014

Village	No. of goats	Adults			Nymphs		Larvae	Total
		AH	RM	RA	A	R		
Athol	10				248	7	81	336
Clare (H1)	10	25	5		267	11	50	358
Clare (H2)	10	1	1		67		17	86
Cortenburg	10	2			221	1	31	255
Gottenburg-C	10	3	2		336	10	60	411
Hlavekisa	10	2	3		378	4	111	498
Hluvukani (H1)	10	2			143		5	150
Hluvukani (H2)	10				72		8	80
Ludlow	10	5			274	47	60	386
Phungue	10	1			150		127	278
Share	10	50	12		128	4	1	195
Seville	10				173		41	214
Thorndale	10	11			247		21	279
Timbavati	10	7	2	1	134	26	70	240
Utah	10	3			123		33	159
Welverdiend	10	2			186		16	204
Total	160	114	25	1	3147	110	732	4129

*Abbreviations*: H1, Household 1; H2, Household 2; AH, *Amblyomma hebraeum*; RM, *Rhipicephalus microplus*; RA, *Rhipicephalus appendiculatus*; A, *Amblyomma*; R, *Rhipicephalus*

In 2013, 3387 ticks were collected from 117 goats in July and a further 2732 ticks from 184 goats in November (Tables 1, 2). In 2014, 2884 ticks were collected from 169 goats in March, and 4129 ticks were removed from 160 goats in July (Tables 3, 4). Large numbers of *A. hebraeum* nymphs were present all year round (Tables 1, 2, 3 and 4). Nymphs (*n* = 7537) were either *A. hebraeum* or belonged to the genus *Rhipicephalus*, which were not further identified to the species level. The relative proportion of nymphal *Amblyomma* ticks *versus* other tick species was 93.5%. The mean number of adult *A. hebraeum* ranged between 0.2 and 2.8 ticks per goat, whereas nymphs ranged between 7.5 and 19.7 ticks per goat (Table 5). There was a significant difference in the mean burden of ticks per goat between the four collections (Table 5). The ratio between adults and nymphs ranged from 1:2.7 in summer to 1:55.1 in winter (Table 5).

**Table 5:** Mean number of *Amblyomma hebraeum* ticks per goat collected in each sampling period

Period	*Amblyomma hebraeum*		Ratio adult:nymph
	Adults	Nymphs	
July 2013	0.2	10.8	1:55.1
November 2013	2.8	7.8	1:2.7
March 2014	1.3	7.5	1:5.8
July 2014	0.7	19.7	1:27.6

*Notes*: A highly significant (*P* < 0.01) difference of *A. hebraeum* adults per goat was found between July 2013 and November 2013 (*U*_(93)_ = 3, *Z* = 4.93424, *P* < 0.00001); between November 2013 and March 2014 (*U*_(93)_ = 69.5, *Z* = 2.73941, *P* = 0.00614); between November 2013 and July 2014 (*U*_(86)_ = 30, *Z* = 3.91612, *P* = 0.00008); and between July 2013 and March 2014 (*U*_(87)_ = 36, *Z* = -3.7199, *P* = 0.0002). A highly significant (*P* < 0.01) difference of *A. hebraeum* nymphs per goat was found between November 2013 and July 2014 (*U*_(86)_ = 32.5, *Z* = -3.82986, *P* = 0.00012) as well as between March 2014 and July 2014 (*U*_(81)_ = 28, *Z* = -3.87236, *P* = 0.0001)

### Pathogen detection

**Fig. 3:**
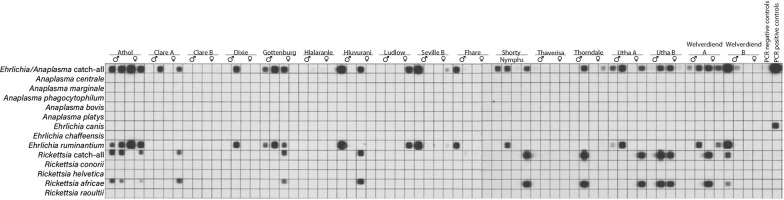
Reverse line blot hybridisation on adult *Amblyomma hebraeum* ticks collected in November 2013 from goats in the Mnisi Community Area

**Fig. 4:**
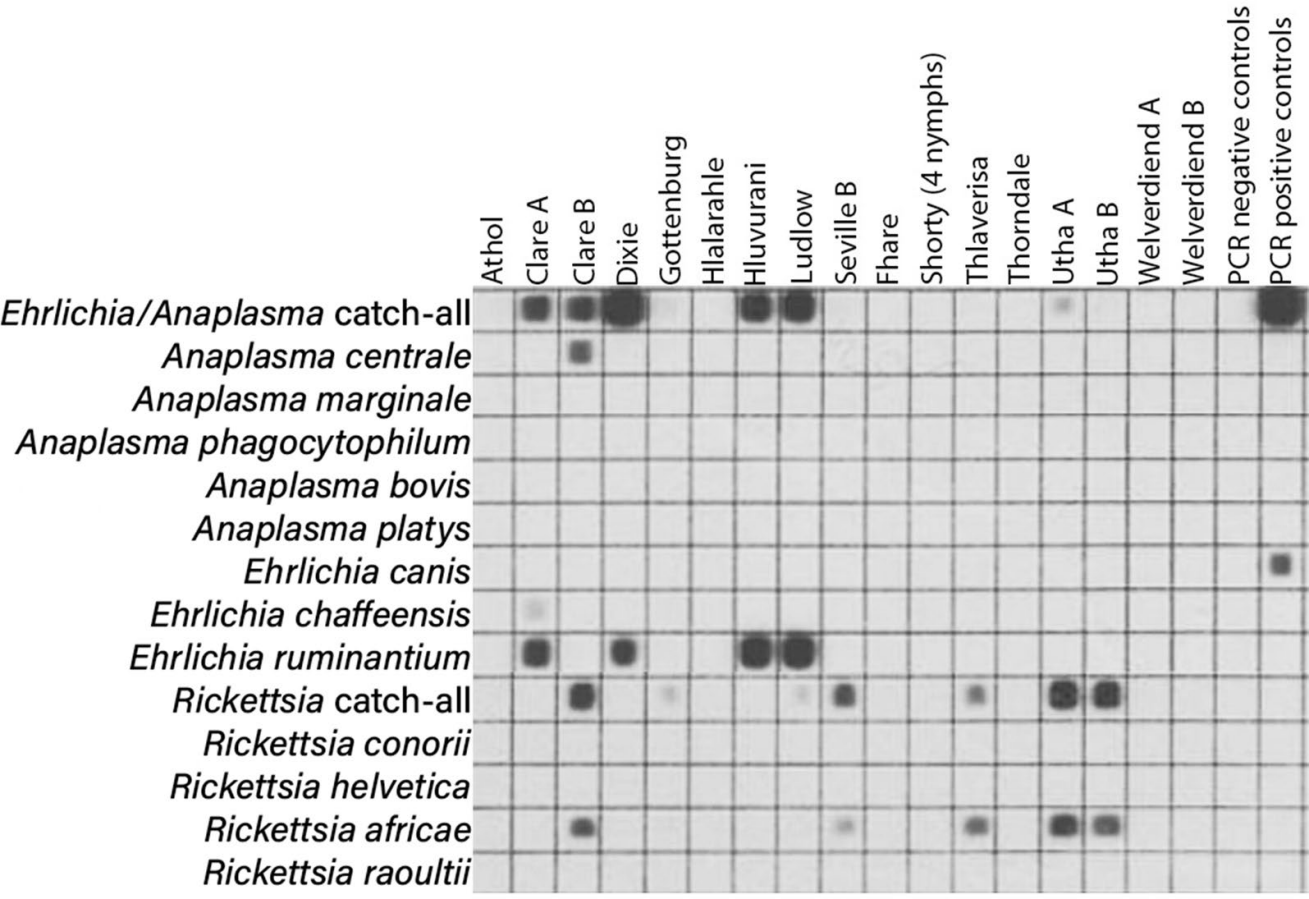
Reverse line blot hybridisation on nymphal *Amblyomma hebraeum* ticks collected in November 2013 from goats in the Mnisi Community Area

The reverse line blot results with adult *A. hebraeum* ticks collected in November 2013 are shown in Fig. 3. Adult ticks were positive for either *E. ruminantium* or *R. africae* or both, whereas none of the other rickettsial pathogens were present. The RLB results for nymphs collected in the same month are shown in Fig. 4. They were also clearly positive for either *E. ruminantium* or *R. africae* or both, whereas one *R. africae* positive tick was also positive for *A. centrale* (Fig. 4). The mean prevalence of infection for *E. ruminantium* detected by PCR/RLB in adult *A. hebraeum* ticks was 17.4% (31/178), whereas 15.7% (28/178) were infected with *R. africae* (Table 6). In pooled nymphs, 28.4% were infected with *E. ruminantium*, and 38.8% carried an infection of *R. africae*. Co-infections of *E. ruminantium* + *R. africae* in adult and pooled nymphal ticks were 3.9% (7/178) and 10% (10/67), respectively (Table 6).

**Table 6:** Co-infection of *Ehrlichia ruminantium* and *Rickettsia africae* in adults and nymphal *Amblyomma hebraeum* ticks

Sampling period	Adults				Nymphs			
	No. tested	ER (%)	RA (%)	CI (%)	No. tested pools	ER (%)	RA (%)	CI (%)
July 2013	23	3 (13.0)	1 (4.3)	1 (4.3)	17	2 (11.8)	3 (17.6)	0
November 2013	68	17 (25.0)	16 (23.5)	5 (7.3)	17	4 (23.5)	5 (29.4)	0
March 2014	51	5 (9.8)	5 (9.8)	0	17	7 (41.2)	10 (58.8)	6 (35.9)
July 2014	36	6 (16.7)	6 (16.7)	1 (2.8)	16	6 (37.5)	8 (50.0)	4 (25.0)
Total	178	31 (17.4)	28 (15.7)	7 (3.9)	67	19 (28.4)	26 (38.8)	10 (14.9)

*Abbreviations*: ER, *Ehrlichia ruminantium*; RA, *Rickettsia africae*; CI, co-infection

## Discussion

The rationale for conducting this study was to collect important baseline information concerning ticks infesting goats and possible zoonotic risks as a basis for subsequent sustainable intervention strategies. The study, conducted in the Mnisi Community Area of Mpumalanga Province in South Africa, demonstrates that ticks affected indigenous goats owned by emerging farmers and were also a potential risk of transmitting zoonotic diseases to humans. More than 13,000 ticks were collected from goats distributed over 17 different households, with *A. hebraeum* being the predominant species collected, followed by *R. microplus*. Small numbers of *R. appendiculatus*, *R. simus* and *R. zambeziensis* were also identified (Tables 1, 2, 3 and 4). With only one species of *Amblyomma* found here, it can be assumed that all *Amblyomma* nymphs also belonged to *A. hebraeum*. However, this cannot be assumed for *Rhipicephalus* nymphs, since there were adult ticks of at least four different *Rhipicephalus* species identified. As far as larvae are concerned, these consisted of *Amblyomma* as well as *Rhipicephalus*. Although not individually identified, the presence of *Amblyomma* larvae was confirmed in each of the four collections. Thus, all three developmental stages of *A. hebraeum* were collected at each sampling, whereby the ratio between adults and nymphs ranged from 1:2.7 in summer to 1:55.1 in winter (Table 5). Although a considerable number of goats (*n* = 630) was sampled, the number of collections was limited to only four, which implies that the protocol was not designed to reveal the entire seasonal dynamics. Instead, the study was designed to determine the species distribution and abundance of ticks on goats at different times of the year as a baseline for subsequent intervention strategies.

The seasonal occurrence of *A. hebraeum* has been studied before and appears to be climate-dependent and to vary throughout its distributional range of the tick. The species has a three-host life-cycle, with larvae, nymphs and adults feeding on separate hosts. In general, adults tend to be most numerous during the warm, wet summer months, larvae during the colder, dry, late autumn and winter months, and nymphs during the winter and spring months. Our results confirm that varying numbers of all stages of development can often be found on hosts throughout the year [20]. In the warm, moist, lowveld regions of Mpumalanga Province, the life-cycle seems to be continuous, with little indication of a definite seasonal pattern of abundance for the various life stages.

A previous study conducted in Mpumalanga Province between 1991 and 1993, wherein ticks were collected from indigenous goats owned by small-scale farmers, confirmed that *A. hebraeum* was the most common species, followed by *R. appendiculatus* and *R. evertsi* [21]. A slightly different situation with respect to species composition on domestic goats was reported from Zimbabwe, where *R. evertsi* was the predominant species [22]. From the neighbouring Maputo province in Mozambique, a similar species composition has been reported, with *A. hebraeum*, *R. appendiculatus*, *R. microplus*, *R. evertsi* and *R. simus* [11].

The finding of *R. microplus* on goats is in agreement with records reported in earlier studies [16]. Nyangiwe & Horak [15] concluded that *R. microplus*, which was considered to be a cattle tick, is in the process of adapting to goats. This implies that these ticks successfully completed their life-cycle on the goats and could even do this in the absence of infested cattle [15]. Therefore, although domestic cattle are the most efficient hosts of *R. microplus*, goats also play a significant role as host. Since only *R. microplus* was recovered from the goats, and *R. decoloratus* was not found, this may suggest that *R. microplus* has displaced *R. decoloratus*, which is in accordance with previous studies [16, 23]. However, *R. decoloratus* is also a cattle tick as *R. microplus,* and not finding *R. decoloratus* on goats does not prove displacement.

Tønnesen et al. [23] and Horak et al. [11] discussed several reasons for the displacement of *R. decoloratus* by *R. microplus*. One of the reasons was that *R. microplus* males mate with female *R. decoloratus*, leading to the production of sterile offspring [23, 24]. An additional range expansion of *R. microplus* has more recently been reported in South Africa, where the tick is now present throughout the coastal region of the Eastern Cape Province and at multiple localities of the Western Cape Province [25]. Interestingly, however, *R. microplus* has not replaced the indigenous species *R. decoloratus* at all localities for reasons to be further investigated [26].

The mean prevalence of infection for *E. ruminantium* by PCR/RLB in adult ticks was 17.4%, while 28.4% of the pooled nymphs were infected. The relatively high prevalence in nymphs was probably due to pooling of nymphs into groups of 10, which increased the chances of detecting *E. ruminantium*. The prevalence of *E. ruminantium* in adult *A. hebraeum* collected from goats in the Mnisi area falls within the same prevalence range recorded in *A. hebraeum* in other studies conducted in South Africa and Zimbabwe [27, 28]. More recently, however, a much higher *E. ruminantium* prevalence of 68% in *A. hebraeum* ticks collected from goats in four different provinces, including Mpumalanga, was reported [29]. Moreover, *E. ruminantium* has been detected in blood samples collected from goats in KwaZulu-Natal and the Free State provinces, with an infection prevalence of 16.4% and 4.2%, respectively [30].

In the Mnisi area, clinical cases of heartwater have been suspected for some time in goats which died after walking in a circle, paddling with their legs and with a protrusion of the neck. This is most likely due to *E. ruminantium* infection in the brain, which requires confirmation by the detection of rickettsial inclusion bodies in endothelial cells of capillaries in the brain. If confirmed, it also indicates that indigenous goat breeds are more susceptible to heartwater than suggested [4]. Certain breeds of goats are more resistant than others, making the introduction of high-producing animals into rural *Amblyomma* areas difficult [21]. Moreover, trade and movement of livestock across geographical regions jeopardise the establishment of a robust immunity against heartwater, for instance in restocking exercises in Mozambique [31].

*Rickettsia africae* infections were also detected in adult *Amblyomma* ticks (15.7%), whereas nearly 40% of pooled nymphal samples carried this infection. Co-infections of *E. ruminantium* and *R. africae* in adults and nymphal tick pools occurred in 3.9% (7/178) and 14.9% (10/67), respectively. African tick-bite fever has been detected in *Amblyomma* ticks and in patients in at least 14 African countries, with up to 11% of infections being acquired by international travellers returning from South Africa [6]. Although exposure of the local community in the Mnisi area to infected ticks must be substantially higher, human cases have not been reported. Elsewhere, for instance in Madagascar, high infection rates of *R. africae* in *A. variegatum* have been correlated with a low prevalence of anti-rickettsial antibodies in healthy pregnant women [32]. A systematic one-health approach encompassing the entire ecosystem, wherein humans, livestock, wildlife and (ecto)-parasites co-exist, is currently underway and is expected to answer the many questions that go beyond the scope of the limited investigation reported here.

Lameness due to predilection of ticks for the interdigital space of feet was observed in 89% of the households. The occurrence of foot abscesses in goats has been linked to the seasonal abundance of adult *A. hebraeum* and *R. glabroscutatum* [14]. Another tick that is notorious with respect to foot infestations and temporary lameness is *Hyalomma rufipes*, in particular on Merino sheep in the Free State of South Africa [33]. Despite the importance of goats for the livelihoods of farmers in the area, tick control is not practiced on any systematic scale. At a recent farmer’s day, clear interest was expressed by the owners for improved animal health management through tick control on their goats, leading to enhanced livelihoods. As a result, and after consultation with the local authorities and farmers’ associations, several interventions were carried out with the aim of reducing the negative impact of ticks on the health of indigenous goats. Since *A. hebraeum* prefers hairless areas in the lower perennial region, at the axillae, genitalia, on the udder and under the tail of cattle, as well as attaching to the inter-digital space of goats and sheep, control may be more effective by targeted application of acaricides. Interventions carried out thus far include weekly treatment of predilection sites with deltamethrin, formulated as a tick grease, and three-weekly topical applications of a combination of amitraz with deltamethrin for the control of ticks on goats. In general, these interventions have successfully reduced tick burdens, but as soon as they were withheld, infestation levels were back to levels prior to treatment (Jongejan et al., unpublished results). Therefore, acaricide-impregnated collars with high and sustained efficacy against ticks on dogs and cats could be considered. However, due to their browsing behaviour, goats may risk losing these collars. Instead, a leg-band close to the proximity where ticks enter the host and close to their predilection site may be a more sustainable solution. This could dramatically improve the health and wellbeing of the indigenous goat population in the Mnisi area. If successful, this type of control could be applied in a much wider area throughout sub-Saharan Africa, where goats are kept under communal grazing conditions in pastures infested by *Amblyomma* ticks.

## Conclusions

*Amblyomma hebraeum* was the predominant tick species on goats in the Mnisi Community area and was infected with *E. ruminantium* and *R. africae*. Moreover, *R. microplus* appeared to be adapted to feed on goats in this area of South Africa. The use of acaricide-impregnated leg-bands is recommended as a sustainable tick control method on goats to kill ticks and reduce lameness. This intervention is probably the most effective one during the summer period at the peak of the adult tick infestation. Finally, the observation that goats are continuously challenged by substantial numbers of *E. ruminantium-*infected *A. hebraeum* ticks throughout the year is a major obstacle preventing the upgrade of local goat breeds. Humans may be at risk of contracting tick-bite fever in this area.

## Data Availability

Data supporting the conclusions of this article are included within the article. The dataset used in this study and the extracted DNA from the collected ticks are available upon reasonable request.

## Abbreviations

RLB: reverse line blot
SDS: sodium dodecyl sulfate
PCR: polymerase chain reaction
streptavidine-POD: streptavidine-peroxidase
SSPE: sodium chloride-sodium phosphate-EDTA
